# Botulinum toxin chemodenervation for childhood strabismus in England: National and local patterns of practice

**DOI:** 10.1371/journal.pone.0199074

**Published:** 2018-06-14

**Authors:** Ameenat Lola Solebo, Anne-Marie Austin, Maria Theodorou, Chris Timms, Joanne Hancox, Gillian G. W. Adams

**Affiliations:** 1 Department of Strabismus and Paediatric Ophthalmology, Moorfields Eye Hospital and University College London Institute of Ophthalmology, National Institute of Health Research, Biomedical Research Centre, London, United Kingdom; 2 Lifecourse Epidemiology and Biostatistics Section, Population, Policy and Practice Programme, University College London Great Ormond Street Institute of Child Health, London, United Kingdom; 3 Ulverscroft Vision Research Group, London, United Kingdom; 4 Clinical and Academic Department of Ophthalmology, Great Ormond Street Hospital, London, United Kingdom; University of Florida College of Medicine, UNITED STATES

## Abstract

**Background:**

Botulinum toxin injection chemodenervation is a well-established intervention for adult strabismus, and has also been recognised as an effective alternative to routine incisional surgery for paediatric disease. We aimed to investigate the temporal patterns of practice, indications and outcomes of chemodenervation for paediatric strabismus at national and tertiary centre level.

**Methods:**

Retrospective study using routinely collected patient data: Hospital Episode Statistics (HES) data were used to identify children undergoing non-incisional strabismus procedures in England from 2007 to 2016. Single–centre retrospective data on children undergoing botulinum toxin injections (Dysport^®^ 2.5 units/ 0.1ml) as an isolated intervention (not involving incisional procedures) was undertaken to identify indications and outcomes. Successful outcome was defined as deviation <11 prism dioptres (PD).

**Results:**

Between 2007 and 2016, there was no increase in the proportion of childhood strabismus involving non-incisional procedures. Amongst 150 children undergoing chemodenervation for strabismus within the tertiary centre, the most common diagnoses were acute onset esotropia (n = 34), infantile esotropia (n = 16) and consecutive exotropia (n = 15). Median age at injection was 8.5 years (range 0.9–15 years), and median follow up 12 months (6 months—11 years). Success rates differed by diagnosis, from 66% (non or partially accommodative esotropia) to 0% (congenital cranial disorders). Adverse events were seen in 62/150, 41%, most commonly transient ptosis (39%, n = 58). Overcorrection was seen in 14/119, 13%. Mild subconjunctival haemorrhage (n = 2) was the only other adverse event.

**Conclusions:**

Botulinum toxin for childhood strabismus has an acceptable safety profile, and considerable potential therapeutic benefit. However, nationally there has been no increased uptake of chemodenervation non-incisional procedures. Further prospective studies are necessary to understand the predictors of outcome within the separate clinical subgroups, to guide clinical decision making.

## Introduction

Strabismus, or squint, a common disorder affecting up to 5% of children,[1] is associated with a significant negative impact on quality of life.[2] Childhood squints are often managed with correction of any refractive error (typically with glasses), and management of any amblyopia with occlusion therapy.[3] Where further intervention is indicated, extraocular muscle surgery remains the gold standard therapy.[4] A significant proportion of those who undergo incisional squint surgery in childhood require further surgery, to correct residual misalignments, or consecutive misalignments in which the direction of the squint is reversed due to secondary post-operative changes in extraocular muscle function.[5] Each subsequent surgery carries a higher risk of poor outcome. [3,5]

Botulinum toxin A (BTXA) injection to the extraocular muscle, and the consequent induction of local paralysis, is an established treatment for adult strabismus.[6] In the first Cochrane systematic review of outcomes following BTXA for strabismus, BTXA injection was also described as equally effective as surgery for children with residual esotropia.[7] Chemodenervation is muscle sparing, and associated with significantly reduced duration of general anaesthesia.[8] Whilst repeated injections are necessary for adults to maintain good outcome, children are in some cases able to maintain the normal alignment gained through BTXA chemodenervation after the temporary paralysis has resolved.[9] It has been used as an adjunct to incisional strabismus surgery [10] and as a secondary therapy to treat consecutive misalignments.[11,12] More recently it has been shown to be a successful primary intervention for childhood acute onset esotropia, and more cost-effective than traditional surgery.[8]

Serious adverse events following BTXA chemodenervation, such as sight-threatening haemorrhage and globe perforation, are rare.[4] The Cochrane review suggested that almost half of all patients were affected by reversible treatment complications such as ptosis, overcorrection and haemorrhage, with higher complication rates following injection in children versus adults.[4] However, only one of the included studies used Dysport^®^ (*abo*boulinumtoxinA, the UK formulation of BTXA) rather than Botox^®^ (*ona*botulinumtoxinA, the American formulation), with evidence that the different forms of BTXA differ in their adverse event profile.[13] Nevertheless, concerns regarding the safety profile of chemodenervation, alongside the uncertainties regarding clinical benefit and dosage, may be acting as obstacles to the adoption of botulinum toxin as a non-incisional alternative in the management of paediatric strabismus.

Routinely collected clinical data can form an accessible repository of the information necessary to understand treatment patterns at regional and national level. The National Health Service Hospital Episodes Statistics (HES) is a centralised database of details on patient activity. Aggregated data on primary procedures undertaken each year within English Hospitals, categorised by gender and age group, are freely available. Individual level data on clinical indications for undertaken procedures, and outcomes for treated children are not freely available from HES. These data are however available through review of hospital casenotes.

The aim of this retrospective population and centre-based UK study was to understand the temporal patterns of the use of botulinum toxin for children undergoing procedures for strabismus, the indications for treatment, the effectiveness for different strabismus indications, and the overall safety profile.

## Methods

### Population based study

In order to understand the temporal patterns of the use of botulinum toxin for paediatric strabismus, the following data were extracted from HES (http://content.digital.nhs.uk/hes): the number of non-incisional strabismus procedures as a proportion of all undertaken procedures (S1 Table) between 2008 and 2016. Strabismus surgery is categorised using the alphanumeric digit codes within the headings C31–C35, and C37. Botulinum toxin chemodenervation procedures are categorised as non-incisional strabismus procedures using C37.8 and C37.9.[14] Within HES data, the freely available aggregated patient data is divided by age into those aged 0–14 years, and those aged 15 years and over. Data on individuals aged 0–14 years were extracted from HES. Data on the diagnoses of children admitted to NHS hospitals for inpatient care, for which the primary cause of admission was a strabismus disorder (S1 Table) were also extracted.

### Centre based study

In order to obtain the data necessary to understand the indications and outcome of chemodenervation in childhood, we undertook a retrospective case series review of all children undergoing botulinum toxin injection to the extraocular muscles for horizontal strabismus between 1994 and 2015 at a UK tertiary referral centre. The appropriate Institutional Review Board (IRB) approvals were obtained, specifically Moorfields Eye Hospital Audit department approval for the execution of a service evaluation study undertaken by clinicians within the service which aimed to provide data on the standards achieved. In adherence with National Health Service guidance on audit and service evaluation studies, the IRB waived the requirement for informed consent. This work adhered to the tenets of the Declaration of Helsinki. All children (aged under 18 years) who underwent injection to the extraocular muscles and had at least 6 months of follow up were eligible. Children given BTXA injection as an adjunct to incisional squint surgery were excluded. Cases were identified through semi-automated searches of institutional electronic patient data management systems with verification through hand searches of institutional paper theatre records.

All children had undergone injection using botulinum toxin A (Dysport^®^, Ipsen Ltd, Maidenhead, England). The standard dose of Dysport^®^ 2.5 units (in 0.1ml of normal saline) was used for each muscle. Prior to injection, topical adrenaline 0.01% was used to improve visualisation of the anterior ciliary vessels as a marker of muscle insertion, and in order to reduce the incidence of conjunctival haemorrhage. Electromyographic monitoring was used to target delivery of BTXA via 25-gauge monopolar injection electrode, which was left in the muscle for 1 minute following BTXA delivery to reduce leakage from injection site.

Data were collected from case notes using a standardised study specific data collection form. Collected data included diagnosis, age at treatment, whether BTXA was intended as a therapeutic intervention or as a post-operative diplopia test (to indicate whether the child would experience diplopia following a further incisional strabismus surgery), time from onset to injection, pre and post-operative angle of deviation, stereopsis at follow up and whether the child had proceeded to surgical intervention.

Criteria used to define outcome: in accordance with the definitions used within the Cochrane systematic review, success was defined as deviation less than 11 prism dioptre (PD) at 4 months following injection. Partial success as deviation less than 21PD at 4 months post injection. Sustained success was that seen at final follow up (ie at least six months after injection). Post-operative ptosis was defined as significant if the lid margin crossed the superior pupillary border as seen in a standard examining room (ie artificial indoor lighting).

Data were collected from case notes using a standardised study specific data collection form. Collected data included diagnosis, age at treatment, whether BTXA was intended as a therapeutic intervention or as a post-operative diplopia test (to indicate whether the child would experience diplopia following a further incisional strabismus surgery), time from onset to injection, pre and post-operative angle of deviation, stereopsis at follow up and whether the child had proceeded to surgical intervention.

Descriptive analyses of outcome were conducted. Statistical analyses were undertaken to investigate the association between the incidence of post-operative ptosis and injection site (Chi^2^ test), and age at botulinum toxin, and between strabismus duration and success with treatment clinical groups with sufficiently large number for the statistical power necessary to undertake such analyses. Univariable and multivariable regression modelling was undertaken. Analyses were conducted using STATA statistical software version 13 (StataCorp, College Station, Texas, USA). 95% confidence intervals are reported where appropriate.

## Results

### National pattern of practice

**Fig 1:**
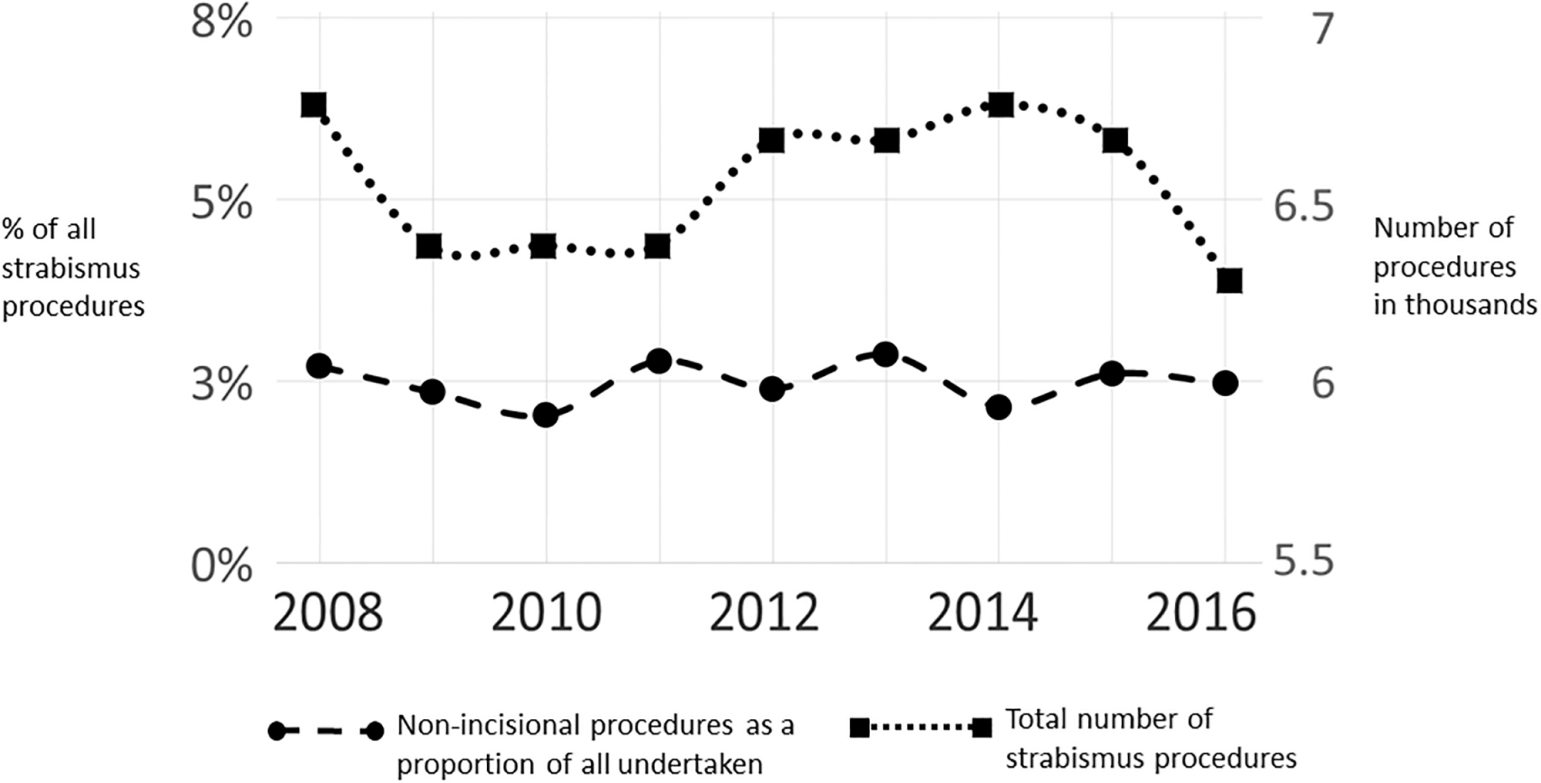
Patterns of practice of strabismus surgery for children aged 0–14 between 2008 and 2016 in NHS England hospitals.

**Fig 2:**
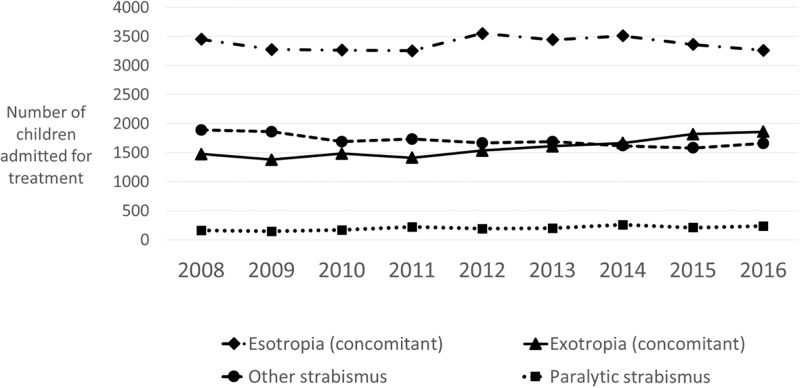
Diagnoses of children admitted to NHS England hospitals for strabismus interventions. Esotropia = convergent strabismus; Exotropia = divergent strabismus; Concomitant = strabismus with equal misalignment in all directions of gaze, ie non-paralytic, non-constrictive, non-restrictive aetiology; Paralytic = associated with extraocular muscle palsy.

In the year ending April 2008, of 6310 strabismus interventions undertaken in children (individuals aged 0–14 years), 2.7% were non-incisional chemodenervation procedures. In the year ending April 2016, of 6067 procedures, 153 (2.5%) were chemodenervation procedures. Between these two time points, there was no significant change in the proportion of undertaken non-incisional childhood procedures (Fig 1). There was also no significant change in the types of strabismus affecting children who were admitted for interventions over this period (Fig 2). The most common diagnosis for children admitted for strabismus intervention was concomitant (ie non paralytic, non constrictive, with equal sized misalignment in all directions of gaze) esotropia (ie horizontal convergent squint).

### Tertiary centre based patterns of practice

150 eligible children were identified (Table 1). 82 children (55%) were female. In 79% (119/150) of children, BTXA was a therapeutic intervention, and in the remaining 21%, it was undertaken as a diagnostic procedure for post-operative diplopia ahead of planned incisional strabismus surgery (Table 1). Median age at injection was 8.5 years (range 0.9 to 15 years), and median follow up was 12 months (range 6 months to 11 years). 95% of children underwent injection under general anaesthesia: the remaining seven children, all aged over 12 years, underwent chemodenervation under topical anaesthesia. The most common indication for chemodenervation was acute onset constant comitant esotropia (ACCE), defined as a newly acquired comitant esotropia in a child in whom lateral rectus under-action has been excluded,[3,8,9] and in whom there is no significant reduction in size (>10 prism dioptre, PD) of esotropia with hyperopic correction. Of these 33 children, 17 children were aged under 5 years at time of ACCE onset, and 11 aged 6–8 years. Time from onset of ACCE to intervention for these 33 children ranged from 1 month to 13.5yrs (median 7 months). 19 children (58%) underwent therapy following esotropia of more than 6 months duration.

**Table 1:** Clinical features of cohort.

Diagnosis	Therapeutic, n (%)	Pre-treatment deviation, median PD (range)	Median age at treatment, yrs (range)	Bilateral recti injection, n (% of therapeutic)	Median follow up, months (range)
**Esotropia**					
*Acute onset esotropia*^a^, *n = 34*	33 (97%)	45 (14–75)	6.9 (0.9–13.5)	26 (79%)	12 (6–43)
*Infantile esotropia*^a^, *n = 16*	10 (63%)	40 (18–80)	0.8 (0.4–13.9)	10 (100%)	11 (7–100)
*Non accom esotropia*^a^, *n = 14*	11 (79%)	40 (8–70)	7.8 (3.9–10.5)	8 (73%)	13 (7–40)
*Consecutive esotropia*, *n = 14*	12 (86%)	25 (6–50)	9.3 (3.8–15.2)	6 (50%)	14 (6–22)
*Residual esotropia*^a^, *n = 9*	8 (89%)	40 (20–75)	10.2 (1.4–13.9)	6 (75%)	13 (6–58)
*Part*. *accom esotropia*^a^, *n = 7*	4 (57%)	40 (20–50)	9.4 (4.8–10.2)	3 (75%)	15 (6–40)
*CCDD*, *n = 6*	5 (83%)	40 (16–80)	8.5 (4.8–15.4)	2 (40%)	20 (8–53)
*Sixth nerve palsy*, *n = 5*	5 (100%)	50 (20–60)	4.4 (2.8–7.8)	3 (60%)	17 (6–28)
*Total*, *n = 112*^b^	92 (82%)	40 (6–80%)	7.2 (0.4–15.6)	81 (88%)	12 (6–100)
**Exotropia**					
*Consecutive exotropia*, *n = 15*	11 (73%)	30 (8–60)	13.8 (9.3–15.9)	11 (100%)	14 (6–22)
*Residual exotropia*^a^, *n = 5*	5 (100%)	20 (18–45)	10.5 (7.2–14.4)	1 (20%)	24 (10–120)
*Constant exotropia*^a^, *n = 5*	2 (40%)	45 (30–60)	11.8 (9.3–13.9)	1 (50%)	9 (6–45)
*Intermittent exotropia*^a^, *n = 5*	4 (80%)	25 (20–40)	9.7 (8–10.7)	4 (100%)	11 (6–21)
*Alternating exotropia*^a^, *n = 3*	2 (67%)	25 (12–40)	11.6 (11.2–12)	2 (100%)	10 (7–13)
*Total*, *n = 38*^b^	27 (71%)	30 (8–60%)	12.6 (7.2–15.9)	12 (44%)	12 (6–120)

Pre-treatment deviation as measured at 33cm

Accom = accommodative

Part = Partially

CCDD = Congenital cranial dysinnervation disorder

^a^Concomitant strabismus

^b^Includes 7 ‘other’ esotropia diagnoses: alternating, congenital, cyclical, and 5 ‘other’ exotropia diagnoses: congenital, secondary, recurrent

### Outcomes by clinical indication

The majority of children who underwent therapeutic BTXA had an esotropia (92/119, 77%). 47 of these children had a successful outcome (ie <11PD, 51%, 95% CI 40%–62%) which was sustained in 41 (45%, 95% CI 34%–55%) (Table 2). Partial success was seen in a total of 81 children who underwent injections for esotropia (88%, 95% CI 80%–94%) and was sustained in 69 (75%, 95% CI 65%–83%). Eight of the 92 children underwent repeated toxin injections (9%, 95% CI 4%– 16%). Thus far, 43/92 children have proceeded to undergo incisional strabismus surgery (47%, 95% CI 36–57%).

**Table 2:** Outcomes following therapeutic chemodenervation, by diagnosis.

Diagnosis	Success		Partial success only^b^		Failure (≥21PD)	Subsequent incisional surgery^c^ (95% CI)
	Early	Sustained^a^ (95% CI)	Early	Sustained^a^ (95% CI)		
Esotropia						
Acute onset esotropia, n = 33	11 33%	10 30% (17–47%)	19 58%	16 48% (33–65%)	7 21% (11–38%)	13 39% (25–56%)
Non accomm / partially esotropia, n = 15	10 66%	8 53% (27–79%)	5 33%	5 33% (15–58%)	2 13% (4–38%)	4 26% (8–55%)
Infantile esotropia, n = 10	4 40%	2 20% (0.2–55%)	4 40%	3 30% (11–65%)	5 50% (24–76%)	4 40% (12–74%)
Consecutive esotropia, n = 12	6 50%	5 42% (19–68%)	6 50%	6 50% (25–75%)	1 8% (2–35%)	5 42% (15–72%)
Residual esotropia, n = 8	3 40%	2 25% (3–65%)	2 25%	3 38% (14–69%)	3 38% (14–69%)	4 50% (15–84%)
Sixth nerve palsy, n = 5	1 20%	1 20% (1–72%)	4 80%	4 80% (38–99%)	0	2 40% (5–85%)
CCDD, n = 5	0	0	4 80%	3 60% (15–95%)	2 40% (12–77%)	2 40% (5–85%)
Exotropia						
Consecutive exotropia, n = 11	5 46%	2 18% (2–52%)	6 55%	4 36% (15–65%)	5 45% (21–72%)	5 45% (17–76%)
Residual exotropia, n = 5	2 40%	0	3 60%	3 60% (15–95%)	2 40% (12–77%)	3 60% (15–95%)
Intermittent exotropia, n = 4	2 50%	1 25% (0.1–80%)	2 50%	2 50% (15–85%)	1 25% (5–70%)	2 50% (7–93%)
Alternating exotropia, n = 2	1 50%	1 50% (1–99%)	1 50%	1 % (1–99%)	1 50% (1–99%)	1 50% (1–99%)
Constant exotropia, n = 2	0	0	1 50%	1 50% (1–99%)	1 50% (1–99%)	2 100% (16–100%)

^a^Sustained = as measured at last follow up (at least six months after injection, and as recorded before any subsequent incisional surgery)

^b^Children meeting the criteria for partially successful outcome (<21PD) but not for successful outcomes (<11PD)

^c^No child with a sustained successful outcome underwent surgery

Accom: accommodative

CCDD: congenital cranial dysinnervation disorders

CI: Confidence interval

The highest sustained success rates (ie <11PD) were seen in children with acute onset, partially or non-accomodative, or consecutive esotropias (Table 2). Successful outcomes were seen in 12 of the 27 children who underwent therapeutic BTXA injections for exotropia (44%). Success was sustained in six children (22%). Almost all children (26, 96%) had a successful or partially successful outcome (ie <21PD), which was sustained in 17 (63%). The median age at botulinum injection for children who failed treatment was 6.5 years, ranging from 0.4 to 15.8. Nine children required repeat toxin injections (33%) and 13 proceeded to incisional surgery (48%). Success rates did not differ significantly across exotropia diagnoses.

63/150 of all children complained of diplopia prior to the intervention. These comprised 53 children with esotropia (47% of total), and ten children with exotropia (26% of total). Within the clinical groups with the highest binocular potential (acute onset esotropia and sixth nerve palsies), 18% and 0% of children had documented binocular function post injection, respectively.

#### Relationship between size of deviation, treatment dose, strabismus duration and successful outcome

For children who underwent injection of both medial recti, or both lateral recti, the median proportional reduction in size of deviation (at near) was 55% (interquartile range or IQR 35%–125%, or overcorrection of 25% of original deviation) for acute onset esotropia, 40% (IQR 12%–90%) for non or partially accommodative esotropia, 54% (IQR 32%–85%) for infantile esotropia, 100% (IQR 40%–150%) for consecutive esotropia, 67% (IQR 48%–72%) for residual esotropia, 80% (IQR 70%–103%) for congenital cranial nerve dysinnervation disorder related esotropias, and 80% (IQR 50%–113%) for consecutive exotropia.

Amongst the clinical populations within the cohort which had more than 5 study subjects, there was no significant association between injecting both or one medial or lateral recti and reduction within deviation size. Within the largest single clinical group, those with acute onset esotropia, success rates were similar for the seven children who had undergone unilateral injection (3/7 children, 43%) and the 26 children who received bimedial injection (8/26, 31%) injection (chi^2^ test p = 0.4). The degree of reduction in size of deviation was also similar for children who had undergone unilateral (median 57%, interquartile range 16%–70%) and bimedial injections (median 55%, interquartile range 35%–125%). Stereopsis was restored in 38% of children who had achieved at least partial success following injection for acute onset esotropia, all of whom had undergone BTXA within 9 months of esotropia onset. BTXA failed in 6 children with acute onset esotropia. For one of these, initial success had been seen at 2 months after first BTXA. At the child and family’s request, repeat BTXA was undertaken, which was successful. For the remaining five children, the final deviation for near was reduced from pre-treatment deviation in two children (by 60% and 78%), unchanged in two children, and greater in one child (by 150%). On univariable linear regression analysis younger age at treatment, in days (R^2^ = 0.23 for younger age in days, p<0.01) and shorter time from onset to treatment, in days (R^2^ = 0.18 for shorter time in days, p = 0.05), were both associated with a larger reduction in the size of the deviation (as a proportion of original deviation size). This study was insufficiently powered to enable robust multivariable testing of the association. Whilst reduction in deviation size was greater with earlier treatment there was no statistically significant association between a successful outcome (<11PD) and intervention within 3 months of onset, or within 6 months of onset.

### Adverse events

Adverse events were seen in 62/150 children who underwent injection (41%), the most common being transient ptosis (n = 58, 39%). 7 of these children (5% of those undergoing treatment) developed a ptosis which occluded the visual axis, including two children who developed bilateral occlusive ptosis, with an associated chin lift. In all cases, ptosis had resolved by 4 months following injection. No child required occlusion therapy to prevent deprivational amblyopia while the post-injection ptosis resolved.

Overcorrection (defined as exotropia greater than 5PD following intervention for esotropia, or esotropia greater than 5PD following intervention for exotropia) was seen in 30/112 children 1 month following therapeutic chemodenervation. This occurred more frequently following injection for esotropia. 29 of 92 children were overcorrected following treatment for esotropia, (32%, 95% CI 22–42%), compared to three of the 37 children with exotropia (8%, 95% CI 0–22%). The frequency of overcorrection following therapeutic injection reduced to 13% (14 children, 95% CI 7–20%) at 2–4 months. At this stage, the median size of overcorrection was 10PD (range 6–50). No child was overcorrected at final follow up. Four children (acute esotropia n = 1, non accommodative esotropia n = 1, residual esotropia n = 2) developed diplopia following therapeutic injection (4%, 95% CI 1–9%), one of whom required occlusive therapy to control symptoms. Vertical deviations were seen in three children, and had resolved in all cases by 4 months. Subconjunctival haemorrhage was seen in two children (2%, 95% CI 0.2–6%). There were no other reported adverse events.

Univariable analyses revealed no association between age at injection and the incidence of post-operative ptosis (Mann Whitney score z = 0.12, p = 0.9). There was also no association between previous squint surgery and the risk of ptosis. For children who underwent uniocular recti injections, ptosis was more common following medial than lateral recti injections (Pearson chi^2^ = 3.89, p <0.05).

## Discussion

Over the last decade, there has been no increase in uptake of BTXA chemodenervation for paediatric strabismus. Botulinum toxin is a safe procedure for children, with a low incidence of visually significant ptosis, and lower incidence of significant adverse events. Injection to the medial recti may be associated with a higher risk of iatrogenic ptosis. Success rates differ by clinical indication, with higher success rates seen in children with partially or non-accommodative esotropia. However, a significant sustained reduction in deviation size can be seen across different strabismus populations.

Our retrospective study draws on a large cohort but is without a standardised process of patient selection and data collection. The absence of a uniform and standardised pre and post injection assessment of binocular function or potential, and relatively small sub-group sample sizes also limits this study’s ability to differentially predict outcomes for the various clinical conditions. Nevertheless, the majority of children in all clinical groups showed a sustained reduction in the size of the deviation. We are unable to assess the impact of selection bias on outcomes following uniocular injection versus bilateral injection, and are thus unable to robustly compare these therapeutic options. Our use of extracted data from the NHS Hospital Episode Statistics is limited by the absence of patient level, centre level, or disease level data, which are not freely available from the data service.

Extraocular muscle injection of diluted purified crystalline botulinum toxin A (BTXA), derived from Clostridium botulinum, a gram-positive anaerobic bacterium and the causative agent for botulism, was first used in 1973, when Alan Scott induced paralysis of ocular motility in monkeys.[15] The paralysis lasted for between 2 weeks and 8 months, with duration partially explained by dosage strength. Currently, two versions of BTXA are in common use for strabismus: Dysport^™^, or *abo*boulinumtoxinA developed in the UK and used in the first large scale case series of BTXA in adult strabismus,[6] and Botox^™^, or *ona*botulinumtoxinA, which is derived from the crystalline formation first used by Scott. Protein load and dilution and therefore diffusion rates vary between the two preparations.[16] Whilst Botox^™^ has a greater potency, the conversion factor is uncertain and lies between 1:3 and 1:4 (Botox^™^: Dysport^™^).[13,16] Since its European approval for use in 1990, Dysport^™^ has been in wider use for the management of strabismus in the UK.[4]

The recently updated systematic review of outcomes following chemodenervation for adult and childhood strabismus concluded that injection had been shown to reduce the angle of deviation by amounts comparable to surgery in cases with potential for normal or near-normal binocular vision, such as acute onset esotropia, sixth nerve palsy and infantile esotropia.[4] It was however difficult to undertake any meaningful meta-analyses, particularly of adverse outcomes of chemodenervation, as different doses and types of BTXA had been used.[4]

The strongest existant evidence for the use of BTXA as a replacement for surgery is in the management of residual esotropia. In a 1998 Spanish randomised controlled trial, there was no significant difference in outcomes versus incisional surgery: however, surgery ‘doses’ and Botox^™^ doses differed within this group, reducing the strength of the findings.[12,17]

A recent non-randomised comparative study has demonstrated the effectiveness of BTXA for acute-onset concomitant esotropia, with success rates at 18 months of 67% following bimedial BTXA injection, versus 58% for children treated with incisional strabismus surgery.[8] Benefits over surgery included reduced duration of anesthesia time hospital stay, and cost-effectiveness. Duration of disease was shorter in the group who underwent BTXA (n = 16, median 3 months esotropia duration) than the surgical group (n = 33 median 6 months).[8] The impact of this difference was not investigated. Our lower success rates may be a reflection of the longer disease duration for children within our cohort. However, we suggest that although prompt intervention gives children with acute onset concomitant esotropia a better chance at achieving binocularity, there is evidence of benefit following intervention even in long-standing cases.

Children with infantile esotropia classically have large angle deviations. Treatment in the first year gives the child their best chance at some degree of binocularity, but approximately a quarter of children with early onset esotropia will have spontaneous resolution of the deviation by age 12 months, thus there is a risk of over-treating children and exposing them to the subsequent risk of poor outcome or need for subsequent surgeries.[3,5] BTXA injection has been used to reduce the deviation size as an adjunct to later incisional surgery, but has also been successful as a primary intervention, and has the benefit of significantly lower rates of overcorrection.[18,19] Although a recent meta-analysis of the published observational studies on outcomes following BTXA for infantile esotropia supported its use,[20] a randomised controlled trial of injection versus incisional surgery would provide the evidence necessary to support clinical decisions for this group.

Paediatric sixth nerve palsy responded well to chemodenervation within our group, with all children showing sustained partial success. Successful outcomes have also been reported following adult chemodenervation for sixth nerve palsy,[4] and supports the hypothesis that children with binocular potential have most to gain following BTXA injection.

Sustained partial success was seen in 40% of children with congenital cranial nerve dysinnervation disorders (CCDD), all of whom had Duanes with esotropia. Previous reports have suggested that this group is unlikely to respond well to chemodenervation, but a more recent support suggested good outcomes following unimedial injection.[21]

In the literature, paediatric chemodenervation has been used ‘almost exclusively’ for those with esotropia.[22] However, within our group considerable reduction in deviation size were also seen in children with divergent squint following injection into lateral recti. In addition, the risk of ptosis appeared lower. In groups at risk of multiple further surgeries, or in whom secondary surgery is challenging due to muscle or tenon contractures, BTXA injection may have valuable role to play in reducing the angle of deviation.

The prevalence of ptosis post BTXA chemodenervation ranges from 20% to 50%, depending on the population studied and the BTXA agent and dose used.[4,8,17] It occurs more commonly following chemodenervation in children,[4] with one posited explanation being the nearer proximity of the recti to the eyelid muscles in the smaller paediatric face. Our findings suggest that medial recti injections carry a higher risk of ptosis than injection into the lateral recti, with almost half of the children undergoing injection to one or both medial recti having some transient ptosis. However, the chance of this ptosis interfering with vision is small. This information should be of use when counselling children and families on potential adverse events following injection. However, we undertook no robust assessment of pre and post-operative lid position or levator palpbebralis / superior recti function, so are unable to undertake a more focused investigation into the determinants of post injection ptosis.

Overcorrection following BTXA has been reported in as many as 56% of children following treatment for esotropia.[8] Our lower rates of overcorrection may be a consequence of the different agent and dose used (Dysport^™^ versus Botox^™^).

## Conclusion

Chemodenervation can be considered as a primary intervention for a variety of childhood strabismus disorders, although there is a differential response by clinical condition. Further prospective studies are necessary to elucidate the predictors of outcome and dosage response within the separate clinical subgroups. This research may enable the adoption of BTXA chemodenervation for affected children, resulting in reduced operative time, reduced exposure to general anaesthetic, and long term protection of extraocular muscle structure.

## Supporting information

S1 Table Supplementary Tables 1A and 1B: Hospital Episode Statistics (HES) codes for strabismus surgery and diagnoses. Codes for non-incisional surgery indicated in bold.
(DOCX)
