# Supplementary material for: Botulinum toxin chemodenervation for childhood strabismus in England: National and local patterns of practice
Source: PLoS One. 2018 Jun 14;13(6):e0199074. doi: 10.1371/journal.pone.0199074 (PMC6001959; doi:10.1371/journal.pone.0199074)
Supplement: S1 Table — Codes for non-incisional surgery indicated in bold. (DOCX) [file pone.0199074.s001.docx]

Supplementary table 1:Hospital Episode Statistics (HES) codes for strabismus surgery and diagnoses

Table 1A. HES codes for strabismus surgery, codes for non-incisional surgery indicated in bold

| C31.1 Combined operations on muscles of eye, Recession of medial & resection of lateral rectus muscle of eye |
| --- |
| C31.2 Combined operations on muscles of eye, Bilateral recession of medial recti muscles of eyes |
| C31.3 Combined operations on muscles of eye, Bilateral resection of medial recti muscles of eyes |
| C31.4 Combined operations on muscles of eye, Bilateral recession of lateral recti muscles of eyes |
| C31.5 Combined operations on muscles of eye, Bilateral resection of lateral recti muscles of eyes |
| C31.6 Recession of lateral rectus muscle and resection of medial rectus muscle of eye |
| C31.8 Combined operations on muscles of eye, Other specified |
| C31.9 Combined operations on muscles of eye, Unspecified |
| C32.1 Recession of muscle of eye, Recession of medial rectus muscle of eye nec |
| C32.2 Recession of muscle of eye, Recession of lateral rectus muscle of eye nec |
| C32.3 Recession of muscle of eye, Recession of superior rectus muscle of eye |
| C32.4 Recession of muscle of eye, Recession of inferior rectus muscle of eye |
| C32.5 Recession of muscle of eye, Recession of superior oblique muscle of eye |
| C32.6 Recession of muscle of eye, Recession of inferior oblique muscle of eye |
| C32.7 Recession of muscle of eye, Recession of combinations of muscles of eye |
| C32.8 Recession of muscle of eye, Other specified |
| C32.9 Recession of muscle of eye, Unspecified |
| C33.1 Resection of muscle of eye, Resection of medial rectus muscle of eye nec |
| C33.2 Resection of muscle of eye, Resection of lateral rectus muscle of eye nec |
| C33.3 Resection of muscle of eye, Resection of superior rectus muscle of eye |
| C33.4 Resection of muscle of eye, Resection of inferior rectus muscle of eye |
| C33.5 Resection of muscle of eye, Resection of superior oblique muscle of eye |
| C33.6 Resection of muscle of eye, Resection of inferior oblique muscle of eye |
| C33.7 Resection of muscle of eye, Resection of combinations of muscles of eye |
| C33.8 Resection of muscle of eye, Other specified |
| C33.9 Resection of muscle of eye, Unspecified |
| C34.1 Partial division of tendon of muscle of eye, Tenotomy of medial rectus muscle of eye |
| C34.2 Partial division of tendon of muscle of eye, Tenotomy of lateral rectus muscle of eye |
| C34.3 Partial division of tendon of muscle of eye, Tenotomy of superior rectus muscle of eye |
| C34.4 Partial division of tendon of muscle of eye, Tenotomy of inferior rectus muscle of eye |
| C34.5 Partial division of tendon of muscle of eye, Tenotomy of superior oblique muscle of eye |
| C34.7 Partial division of tendon of muscle of eye, Tenotomy of combinations of muscles of eye |
| C34.6 Partial division of tendon of muscle of eye, Tenotomy of inferior oblique muscle of eye |
| C34.8 Partial division of tendon of muscle of eye, Other specified |
| C34.9 Partial division of tendon of muscle of eye, Unspecified |
| C35.1 Other adjustments to muscle of eye, Transposition of muscle of eye nec |
| C35.2 Other adjustments to muscle of eye, Lengthening of muscle of eye by muscle slide |
| C35.3 Other adjustments to muscle of eye, Insertion of adjustable suture into muscle of eye |
| C35.8 Other adjustments to muscle of eye, Other specified |
| C35.9 Other adjustments to muscle of eye, Unspecified |
| C37.1 Other operations on muscle of eye, Excision of lesion of muscle of eye |
| C37.2 Other operations on muscle of eye, Freeing of adhesions of muscle of eye |
| C37.3 Other operations on muscle of eye, Biopsy of lesion of muscle of eye |
| C37.4 Other operations on muscle of eye, Repair of muscle of eye nec |
| **C37.8 Other operations on muscle of eye, Other specified** |
| **C37.9 Other operations on muscle of eye, Unspecified** |

Table 1B: HES codes for strabismus diagnoses

| Concomitant esotropia | H50.0 | Convergent concomitant strabismus |
| --- | --- | --- |
| Concomitant exotropia | H50.1 | Divergent concomitant strabismus |
| Paralytic strabismus | H49.0 | Third [oculomotor] nerve palsy |
|  | H49.1 | Fourth [trochlear] nerve palsy |
|  | H49.2 | Sixth [abducent] nerve palsy |
|  | H49.3 | Total (external) ophthalmoplegia |
|  | H49.4 | Progressive external ophthalmoplegia |
|  | H49.8 | Other paralytic strabismus |
|  | H49.9 | Paralytic strabismus, unspecified |
| Other strabismus | H50.2 | Vertical strabismus |
|  | H50.3 | Intermittent heterotropia |
|  | H50.4 | Other and unspecified heterotropia |
|  | H50.5 | Heterophoria |
|  | H50.6 | Mechanical strabismus |
|  | H50.8 | Other specified strabismus |
|  | H50.9 | Strabismus, unspecified |
|  | H51.0 | Palsy of conjugate gaze |
|  | H51.1 | Convergence insufficiency and excess |
|  | H51.2 | Internuclear ophthalmoplegia |
|  | H51.8 | Other specified disorders of binocular movement |
|  | H51.9 | Disorder of binocular movement, unspecified |
